# On the Persistence of Discourse Predictions: The Facilitative Effect of Discourse Markers Diminishes in the Presence of Intervening Material

**DOI:** 10.1162/opmi_a_00203

**Published:** 2025-04-22

**Authors:** Merel C. J. Scholman, Hannah Rohde, Vera Demberg

**Affiliations:** Utrecht University, Institute for Language Sciences, Utrecht, the Netherlands; University of Edinburgh, Department of Linguistics & English Language, Edinburgh, UK; Saarland University, Department of Language Science and Technology, Saarbrücken, Germany; Saarland University, Department of Computer Science, Saarbrücken, Germany

**Keywords:** anticipation, language processing, discourse relations

## Abstract

The current study investigates for how long readers maintain expectations about an upcoming discourse relation. We use the pair of discourse markers *On the one hand* (OT1H) and *On the other hand* (OTOH) to test the facilitative effect of OT1H on the processing of OTOH and the sensitivity of this effect to the presence of intervening material. Results from a story continuation study indicate that intervening material slightly weakens the effect of OT1H on offline representations of the discourse. Results from a self-paced reading and two eye-tracking studies suggest that the presence of intervening material diminishes the facilitative effect of OT1H in online processing. These results support memory-based models of processing by showing that discourse dependencies, while they are built as fine-grained representations, are not unbounded in real-time processing.

## INTRODUCTION

Previous work has shown expectation-driven processing at multiple levels of linguistic structure with an emphasis on anticipation as a fundamental aspect of language processing. An open question is whether the strength of such an expectation is affected by the distance separating the cue that gave rise to the anticipation and the anticipated content itself. Memory-based models of processing assume that sentence processing becomes more difficult with additional material – the intervening material can cause decay or interference of the anticipated content (e.g., Lewis et al., 2006). By contrast, expectation-based accounts assume that the processing cost of a word is a function of its expectancy given the prior linguistic context – the context provided by more intervening material can even serve to strengthen the expectation of an upcoming word or structure (Hale, 2001; Levy, 2008; Levy & Keller, 2013). In other words, an expectation-based account would assume that predictions can remain stable if the intervening material does not add content that gives rise to competing predictions, and that predictions can even be strengthened through new intervening material, if this material supports the prediction (but see Vasishth et al., 2018, who show that effects reported in Levy and Keller (2013) are not replicable.). Here we test the nature of predictions at the discourse level, specifically whether they can be maintained across intervening material that does not disconfirm or reinforce the predictions.

Studies providing evidence for predictive language processing have often focused on sentence processing at the syntactic level. In the domain of syntax, when a dependent element is separated from its head, the second element must still follow within the same sentence for the text to be felicitous. For example, one study closely related to the current paper investigated the facilitative effect of the word *either* on the processing of content following *or* (Staub & Clifton, 2006). The results showed that *either* enabled readers to predict the arrival of a coordination structure, which facilitated processing of this structure when it ultimately arrived – which was bound to happen within the same sentence. The current study looks at predictive language processing at the discourse level, a domain in which dependencies can be much more flexible. Oftentimes, intervening clauses and even sentences can occur before the anticipated element, without the discourse being infelicitous. Moreover, intervening material may not necessarily support or strengthen the prediction since discourse-level dependencies are not as tightly constrained as those at other levels of linguistic structure. Rather, the intervening material may contain content that addresses related discourse goals or that is tangential to those goals, thereby possibly diluting the original pre-activation for a specific discourse dependency. This is illustrated in Passage 1 (taken from Scholman et al., 2017).

(1)  Joseph got a job offer from the Edinburgh Zoo and he’s pondering whether he should take it.
  *On the one hand*, he needs the money that this job will pay, [because he should start paying off his student loans this year. Also, his car needs to be serviced by the end of the month.]
  *On the other hand*, he hates the idea of cleaning out panda cages and lion dens every day.

In this discourse, the marker *On the one hand* (OT1H) generates an expectation for an upcoming contrast dependency between two arguments, here referred to as contrast1 (marked by *On the one hand*) and contrast2 (marked by *On the other hand*). Crucially, intervening material may follow the OT1H-marked contrast1, as is the case with the content in brackets in Passage (4), which intervenes before the OTOH-marked contrast2. Scholman et al. (2017) note that 7% of OT1H-marked instances in their corpus study were followed by intervening sentences before OTOH appeared.^1^

The OT1H∼OTOH pairing therefore lends itself to studying the effect of intervening material on the strength of anticipations: at OT1H, the content of contrast1 is introduced and an expectation for an upcoming contrast2 is generated, yielding an open dependency that a comprehender must maintain while processing any intervening material; then, at the point of encountering OTOH, that open dependency must be retrieved and the OTOH sentence must be integrated in the discourse representation as contrast2. This integration may be more difficult when the dependency spans substantial intervening material. During the processing of intervening material, the representation of OT1H and contrast1, as well as the anticipation of upcoming contrast2 marked by OTOH, will have been subject to decay and interference from new content and competing anticipations. Consequently, retrieving the representation of the contrast dependency and integrating OTOH in the discourse structure may be more difficult the greater the distance, thereby impeding the processing of OTOH. However, a competing hypothesis is that the expectation of OTOH based on OT1H can persist across intervening material, due to the pairing’s strong statistical dependency.

In the current work, we exploit these features of the OT1H∼OTOH pairing to study the generation and maintenance of discourse dependencies. Experiment 1, a story continuation study, investigates whether the pre-activation of OTOH is similarly strong in items with and without the intervening material. The results show that comprehenders expected OTOH to follow after having read OT1H. Crucially, the presence of intervening material led to fewer contrastive continuations, indicating that intervening material has an effect (albeit a small one, in this paradigm) on the anticipation of OTOH. We follow up on the offline story continuation result with two reading time studies, to test whether these effects are also detectable during online processing. Experiment 2 is a self-paced reading study; Experiment 3 is an eye-tracking-while-reading study. They were designed to investigate whether the facilitative effect of OT1H on the processing of OTOH is affected by the presence of intervening material. We expected to find that OTOH is processed faster when OT1H is present compared to when it is absent. The question is whether this facilitative effect is sensitive to distance – does intervening material cause discourse expectations to decay? The results of both studies show that this is indeed the case: the facilitative effect of OT1H diminished in the presence of intervening material. The current work therefore supports memory-based models of processing by showing that discourse processing becomes more difficult with additional intervening material.

## BACKGROUND

The studies we review below provide the context for asking how comprehenders’ anticipatory processes can be cued in discourse and how intervening material may affect these processes. This section first discusses expectations in discourse processing, followed by effects of decay and interference on language processing.

### Expectations in Discourse Processing

Research on discourse has shown that comprehenders make predictions about upcoming relations between the ideas expressed in clauses or sentences, referred to as *discourse relations* (e.g., Asr & Demberg, 2020; Barthel et al., 2022; Crible, 2021; Dery & Koenig, 2015; Hoek et al., 2021; Köhne-Fuetterer et al., 2021; Rohde & Horton, 2014; Scholman et al., 2017, 2020; Schwab & Liu, 2020; Xiang & Kuperberg, 2015; Yi & Koenig, 2021). Comprehenders can predict specific upcoming content or discourse relations based on a discourse connective (e.g., *because, however*) or other discourse relational signals such as implicit causality verbs. For example, Köhne-Fuetterer et al. (2021) found in a visual world paradigm that the presence of the connective *however* in passages such as (2) can quickly reverse comprehenders’ expectations of an expected result (getting something sweet to eat), thereby making an unexpected concession (getting something savoury to eat) expected. Further, ERP results indicated that this mental representation update occurred immediately after encountering the concessive connective *however* (Köhne-Fuetterer et al., 2021).

(2)  Marc fancies a small snack. He feels like having something sweet. [However], he gets…

Schwab and Liu (2020) presented a study closely related to the current work: they investigated processing of marker pairs using the English and German pairs of discourse markers *true/sure…but* and *zwar…aber* (see Example (3)). In a self-paced reading paradigm, the authors showed that processing of the connectives *but* and *aber* was facilitated when they were preceded by *true/sure* and *zwar*: reading times on the German connective *aber* and the region following the English connective *but* were faster when these connectives were preceded by their corresponding lexical counterpart compared to when they were not.

(3)  James likes to run outdoors. [**True**, ∅] he has a treadmill in the living room, **but** he often jogs in parks.

Note, however, that even though *true/sure…but* do allow for flexibility in when the second argument will appear in the discourse, these markers consistently occurred together in the same sentence in Schwab and Liu’s (2020) study. Their results therefore cannot speak to the duration for which such facilitative effects can persist.

In the current work, we focus on whether the facilitative effect of discourse markers is affected by the length of the dependencies; specifically, whether comprehenders can maintain discourse predictions across intervening content. This study builds on Scholman et al. (2017) and Scholman et al. (2024b), who investigated discourse predictions based on the discourse markers *On the one hand* (OT1H) and *On the other hand* (OTOH). The marker OT1H signals to the comprehender that a contrast2 will follow, which can facilitate processing of the marker signalling contrast2. Indeed, Scholman et al. (2024b) found faster reading times on OTOH when it was preceded in the previous sentence by OT1H. This effect was visible in early processing (first pass) as well as later processing measures (regression path and total fixation duration). These results establish expectation-driven effects at the discourse level, showing comprehenders’ awareness of the discourse dependency established by a discourse marker.

While Scholman et al.’s (2024b) study contained materials in which contrast1 and contrast2 consisted of two adjacent sentences with only a main clause, contrast2 does not need to follow contrast1 immediately (i.e., intervening content can occur), and contrast2 need not be marked overtly with OTOH. This latter observation was exploited in Scholman et al. (2017) to test whether comprehenders build and maintain predictions of discourse dependencies. They presented readers with passages containing different types of intervening material: In (4), the content of the global contrast condition plausibly contrasts with the content of the OT1H-sentence and thus satisfies the contrast1∼contrast2 pairing; the content of the local contrast condition most plausibly contrasts with information embedded in the subordinating *because*-clause that directly precedes it and thus does not fully satisfy the contrast1∼contrast2 pairing; condition (iii) provided a baseline.

(4)  **Intro:** Joseph got a job offer from the Edinburgh Zoo and he’s pondering whether he should take it.

**OT1H:** *On the one hand*, he needs the money that this job will pay, *because* he should start paying off his student loans this year.

(i)  **Global contrast:** But he could keep looking for a nicer, better-paying job.
(ii)  **Local contrast:** But the loans could be deferred for a few more months.
(iii)  **No contrast:** Also, his car needs to be serviced by the end of the month.

**OTOH:** *On the other hand*, he hates the idea of cleaning out panda cages and lion dens every day.

Scholman et al. (2017) showed that participants can generate fine-grained predictions of upcoming discourse dependencies based on OT1H: in a story continuation and eye-tracking study, they found evidence that the intervening sentence in the local contrast condition did not satisfy the contrast2 expectation, whereas the intervening sentence in the global contrast condition did satisfy this expectation, thus leading to longer reading times on OTOH. Hence, readers not only built, but also maintained, fine-grained predictions of upcoming contrast relations based on OT1H. Crucial for the purpose of the current study: participants were able to maintain these discourse expectations about upcoming dependencies across sentences.

However, Scholman et al. (2017) only presented evidence regarding whether the prediction of a discourse dependency had been satisfied by intervening material. Their data did not speak to the facilitative effect of OT1H on the processing of OTOH in the presence of intervening material, since they did not vary the presence of OT1H. In the current study, we therefore extend prior work to investigate the effect of intervening material on discourse expectations of specific lexical items. We compare production of and reading times on OTOH in a condition with and without OT1H presented before OTOH. We expect to find a facilitative effect of OT1H on the processing of OTOH (similar to effects found Scholman et al., 2024b). In the following section, we review literature on how intervening material can affect language processing.

### The Effect of Decay and Interference on Language Processing

The studies discussed in the previous section have established that comprehenders can make discourse predictions. The next question that arises is how long these predictions can be maintained. Research regarding the timeline of predictions in language processing have focused more on when predictions are made than on how long predictions can persist. We therefore turn to other fields of language processing to understand how intervening material can interfere with processing.

During language processing, lexical and semantic information is temporarily stored in memory, where it can be accessed for retrieval when needed. The ease of retrieval depends on how strong the *memory trace* of the stored information is. The strength of this memory trace can be affected by material that intervenes between the moment that the information is stored and the moment that this information needs to be retrieved – that is, the memory trace can *decay* with the passage of time.

The decay of information has been investigated extensively within the context of models of short-term memory. The classic demonstrations of decay are reported in the Brown-Peterson studies (Brown, 1958; Peterson & Peterson, 1959; see Ricker et al., 2016, for a review of decay studies). Peterson and Peterson (1959) short focused specifically on the effects of interval duration on decay. Participants were given a letter trigram to remember, followed by a retention interval varying from 3 to 18 seconds. During the retention interval, participants were required to count backward by threes in order to prevent rehearsal of the memorandum. Following the retention interval, participants were asked to recall the item from memory. The results showed that performance declined as retention intervals increased. The authors attributed this decline to increasing decay of the memory trace with increasing time.

This interpretation has been debated in subsequent work because the results might also be explained by *interference* (see, e.g., Berman et al., 2009; Underwood & Keppel, 1962). Interference occurs when the comprehender devotes attention to processing the intervening, irrelevant material, which causes the target material to become less available. Since the Brown-Peterson studies, various researchers have attempted to elucidate the effect of interference versus decay on memory and sentence comprehension, and have generally found support for interference effects over decay effects, although some found distinct effects of both (e.g., Berman et al., 2009; Lewandowsky & Oberauer, 2008; Lewandowsky et al., 2009; McKone, 1995, 1998; Oberauer & Lewandowsky, 2014; Reitman, 1974; Van Dyke & Lewis, 2003).

For the current study, we remain agnostic as to whether a possible effect of intervening material should be attributed to decay or interference. Instead, we use the term *deterioration* to encompass both a linguistic (i.e., interference) and purely temporal effect (i.e., decay). We return to this issue in the General Discussion.

Deterioration of information has also been discussed in terms of dependency locality effects (e.g., Gibson, 1998; Lewis et al., 2006): increasing the distance between words linked in syntactic dependencies, such as subject-verb dependencies, results in processing difficulties at the point of retrieval of the second element of the dependency – for example, the distance between *nurse* and *supervised* in (5).

(5)  The administrator who the nurse who was from the clinic supervised scolded the medic.

Such locality effects have been observed in ambiguous structures such as garden path reanalysis (e.g., Ferreira & Henderson, 1991; Van Dyke & Lewis, 2003) as well as in unambiguous structures, such as subject-extracted relative clauses (Grodner & Gibson, 2005) and subject-verb dependencies (Bartek et al., 2011).

Based on evidence from the decay and interference literature, as well as the dependency locality literature, we would expect to find that OTOH is read slower when following intervening material. However, some studies have shown that locality effects can only be detected when the expectation for the predicted element is weak, and not when the expectation is strong (Husain et al., 2014, see also Campanelli et al., 2018; Nicenboim et al., 2015; Stone et al., 2020, but see Safavi et al., 2016). Hence, it is possible that strong expectations can override the decay or interference effect of intervening material by keeping the anticipated material active in working memory. If this is indeed the case, we should expect to find that intervening material does not affect the strength of the facilitative effect of OT1H, given that there is a strong dependency between OT1H and OTOH.

Further, previous work has shown counterexamples to locality effects (see, e.g., Nakatani & Gibson, 2010; Schwab et al., 2022; Vasishth & Lewis, 2006). For example, Konieczny (2000) found that the reading time of the clause-final verb *begleitet* (‘escorted’) in (6) was shorter with a larger number of arguments preceding it, a so-called *anti-locality effect*.

(6)  Er hat den Abgeordneten (an das große Rednerpult) begleitet, und…
  *He has the delegate (to the big lectern) escorted, and…*
  *‘He has escorted the delegate (to the big lectern), and…’*

Anti-locality effects support expectation-based models: interposing elements between arguments and heads do not necessarily interfere, and might even facilitate processing. The effect that intervening material has on predictions depends on the content of the intervening material: the material can only serve to strengthen the prediction if the content contributes to, or re-activates the prediction. Such strengthening may arise if the intervening material provides elements, e.g., arguments of a verb, that saturate the possibilities of what content could plausibly intervene and thus render the predicted element more “inevitable”. If the intervening material gives rise to competing expectations, expectation-based accounts would also predict that the material would weaken the original predictions and thereby not facilitate processing of the originally predicted material. In the current study, the intervening material consists of a clause marked by *because*, which supports the argument made in contrast1. We therefore do not expect the clause to specifically strengthen (no re-activation of the contrast expectation) or weaken the contrast prediction (no strong competing predictions). Hence, in the context of the current study, an expectation-based account would likely predict no effect of the intervening material.

### Current Study

In sum, the current study exploits the flexibility afforded by the OT1H∼OTOH dependency by testing the effect of intervening material on discourse predictions. Experiment 1, a story continuation study, investigates whether the pre-activation of OTOH is similarly strong in items with and without the intervening material. Based on earlier findings, we expect to find an effect of OT1H, with a larger proportion of contrastive continuations in the OT1H-present condition than the OT1H-absent condition. An open question is whether intervening material modulates the effect of OT1H – given that participants can reread earlier materials and thereby re-activate the OT1H-cue, the effect size of the intervening material might be small.

We then present self-paced reading (Experiment 2) and eye-tracking-while-reading studies (Experiment 3 and 4), to test the effect of the OT1H-cue and intervening material on online processing. Based on previous research showing facilitated processing of a second marker in the presence of a first marker (Scholman et al., 2024b; Schwab & Liu, 2020), we expect to find a facilitative effect of OT1H on the processing of OTOH in the absence of intervening material.

A memory-based account would suggest the facilitative effect of OT1H on OTOH to be diminished in the presence of intervening material. Finding a diminished effect could mean that the facilitative effect is weaker compared to when intervening material is absent, or that no effect of OT1H in the presence of intervening material can be found at all. Such a memory-based account is supported by evidence from the decay and interference literature, and would be in line with evidence for locality effects.

By contrast, an expectation-based account of processing would suggest that we should find no effect of intervening material if readers generate predictions of OTOH based on OT1H. In other words, the facilitative effect of OT1H on the processing of OTOH should be equally strong in cases when intervening material is absent and when it is present. This would be in line with anti-locality effects and would extend the domains for which evidence has been found to substantiate expectation-based accounts over memory-based accounts of processing.

## EXPERIMENT 1: STORY CONTINUATION STUDY

A story continuation study was conducted to test whether the pre-activation of OTOH based on OT1H is affected by the presence of an additional clause attaching to contrast1. We asked participants to write a story continuation (one or two sentences) to passages that introduce contrast1 but lack OTOH and contrast2. The continuation task provides insight into comprehenders’ anticipatory processes by asking participants to guess what content is coming next.

### Participants

A total of 162 native English speakers (age range 19–74; mean age 37; 119 female) participated in this study. All participants were registered as workers on the Prolific crowdsourcing website and received monetary compensation for their participation (1.20 GBP).

### Materials

The experimental stimuli used across the three studies reported in this paper consisted of 16 three-sentence passages as in (7), in a 2 (OT1H absent vs. present) × 2 (Intervening Material absent vs. present) design. Each item consisted of an introductory sentence, followed by contrast1.

In the OT1H-cue condition, contrast1 was overtly marked by OT1H; in the no-cue condition, contrast1 was left unmarked. In the Intervening Material-present condition (conditions (i) and (ii) in (7)), OT1H contained a subordinate clause marked by *because*. This clause provided a reason for the perspective presented in contrast1 and was on average 10 words in length. We chose to manipulate presence of a single clause because it presents a discourse structural change by introducing a new discourse dependency, but it only presents a moderate linguistic interval.

(7)  Ronan is thinking about quitting his job at the supermarket after working there for five years.
  (i)  On the one hand, he thinks he could get a more promising job at a multinational, because he studied accounting in college. *[+OT1H,+IM*]
  (ii)  He thinks he could get a more promising job at a multinational, because he studied accounting in college. *[−OT1H,+IM*]
  (iii)  On the one hand, he thinks he could get a more promising job at a multinational. *[+OT1H,−IM*]
  (iv)  He thinks he could get a more promising job at a multinational. *[−OT1H,−IM*]

Note that 16 experimental items is a relatively small number of items for a 2 × 2 design. We opted to present a larger sample of participants with a smaller number of items to address the risk of repeated exposure effects: the OT1H and OTOH are quite heavy and salient in these short prompts. It is possible that repeated exposure to items with OTOH will lead participants to adapt to this marker, and thereby also adapt their expectations. Repeated exposure to *a priori* unexpected structures (such as OTOH in the absence of OT1H or in the presence of intervening material) can reduce or even completely diminish their processing disadvantage, thereby obscuring the effect of interest (cf. Fine et al., 2013).

### Procedure

The experiment was hosted on Lingoturk (Pusse et al., 2016) and distributed via Prolific. The materials were divided into four lists, with the intervening condition being a between-participant variable. Each participant saw one version of 8 experimental and 10 filler items. They therefore only saw the marker OT1H four times throughout the experiment – this was done to mitigate the risk of participants becoming aware of the study’s manipulation. Filler items consisted of short stories similar to the experimental items, without contrast discourse relations or any OT1H or OTOH markers. Each version of every item was completed by ten or eleven people. Participation took 12 minutes on average.

### Analysis Procedure

The continuations were manually annotated for the presence of contrast2 (i.e., whether the content of the continuation provided a contrast with the OT1H clause) and an explicit marker (i.e., whether a connective was used, and if so, which one). To determine whether the continuation presented contrast2, annotators were instructed to test whether the continuation could be expressed using OTOH; if so, this would be a clear indication of contrast2’s presence in the continuation. A random subset of 100 continuations (50 per condition) was double-coded by a second, independent coder who was naive to the purpose of the current study. The agreement between the coders was high: 96%, Cohen’s *κ* = 0.92.

The data were modeled using a Bayesian Bernoulli regression with a logit link, using the brms package Bürkner (2017) in R. The response variable was whether consisted of a contrastive relation or not. The conditions for OT1H and Intervening Material (IM) were deviation-coded (no OT1H, intervening present: both −0.5) and included as fixed effect along with their interaction. Participants and items were specified in the models as random effects, along with variance-covariance matrices. A regularizing LKJ(2) prior was specified on the random effects correlation to ensure that extreme correlations are downweighted (Lewandowski et al., 2009).

We specified principled, weakly informative priors (*N*(0,0.75)). Four chains were run using 10,000 iterations, including 2,000 warm-up, to ensure proper convergence. Model diagnostics were checked using trace plots and R-hat values, which all indicated satisfactory convergence, with R-hat values below 1.01 for all parameters. We report posterior estimates and 95% credible intervals (CRI).

The Bayes factor quantifies the support for or against a model with the effect of interest over another model without the effect of interest. Table 1 provides a guideline for interpreting the Bayes factor, following Lee and Wagenmakers (2014). An important feature of the Bayes factor is that it is sensitive to the prior distribution of the effect of interest – mildly informative priors can strongly bias the Bayes factor. Following state-of-the-art recommendations for Bayes factor computation, we therefore computed the Bayes factor using bridge-sampling tools in brms for three types of informative priors on the parameter representing the interaction term (an approach similar to Mertzen et al., 2024): (1) a more informative prior *N*(0,0.50), (2) a wider prior *N*(0,1.5), and (3) a directional, empirical prior *N*(0.5,0.5). The more informative prior *N*(0,0.50) would tell us the evidence for or against the effect under an a priori belief that the effect size is relatively small. By contrast, the wider prior *N*(0,1.5) would yield evidence for or against the effect under an a priori belief that the effect size can be larger. Both priors allow the direction of the effect to be positive or negative. We also consider a directional prior that matches the estimated range of effects. To derive the empirical priors, we obtained model fits for data from an unpublished study using similar materials and experimental paradigm in Dutch. This prior would tell us whether we have evidence for the interaction, assuming a priori that the interaction is positive in sign.

**Table 1.** Guidelines for the interpretation of the Bayes factor. The order of 1 and 0 in BF_10_ indicates that we look at support for Model 1 over Model 0.

Bayes factor (BF_10_)	Interpretation
> 100	Extreme evidence for M1
30–100	Very strong evidence for M1
10–30	Strong evidence for M1
3–10	Moderate evidence for M1
1–3	Anecdotal evidence for M1
1	No evidence
1/3–1	Anecdotal evidence for M0
1/10–1/3	Moderate evidence for M0
1/30–1/10	Strong evidence for M0
1/100–1/30	Very strong evidence for M0
> 1/100	Extreme evidence for M0

### Results

**Figure 1.**
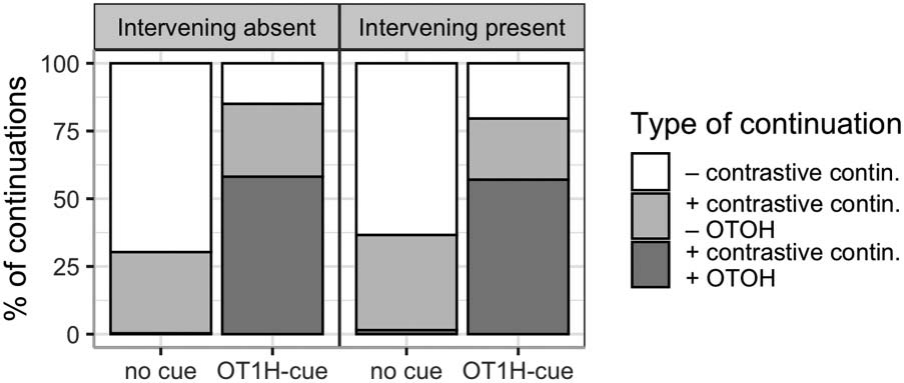
Percentage of contrastive continuations per condition, Exp. 1.

Figure 1 presents the percentage of contrastive continuations (with and without the participants’ explicit usage of OTOH) per condition.

#### Proportion of Contrastive Continuations.

**Figure 2.**
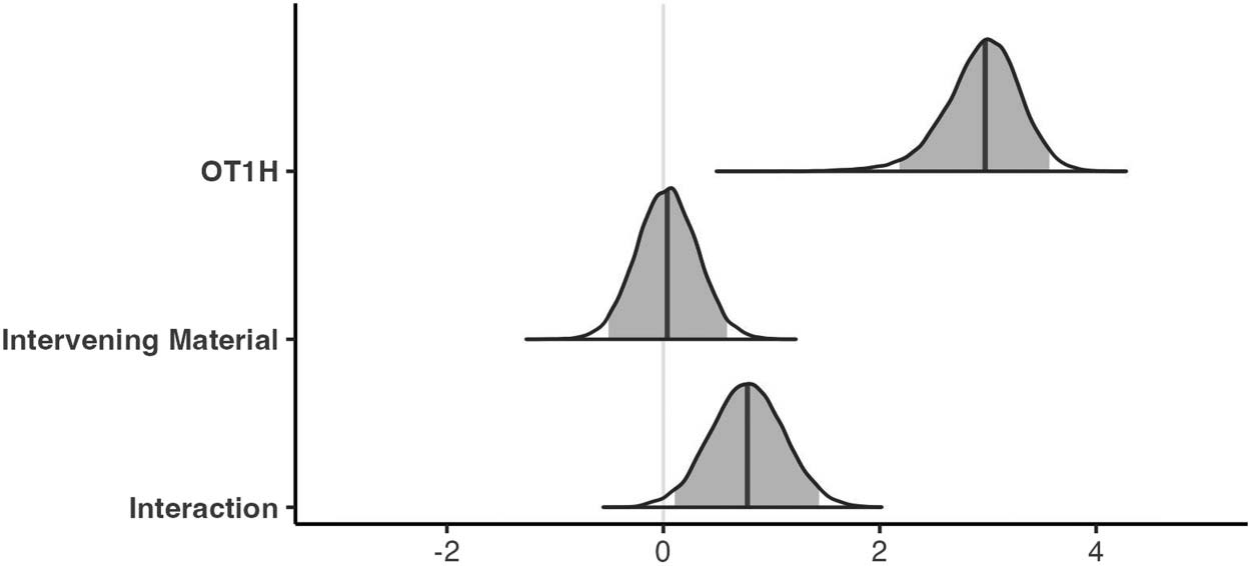
Posterior distributions for the comparisons between conditions, Exp. 1. The darker line shows the median posterior effect estimate; the light gray area indicates the 95% credible interval.

Posterior distributions for the estimated parameters are depicted in Figure 2; the model results are shown in the Appendix (Table A1). As expected, the analysis indicated that the OT1H-cue condition yields more contrastive conditions than the no-cue condition ($\hat{\beta }$ = 2.95, 95% CRI = (2.18, 3.57)). There was no main effect of Intervening Material ($\hat{\beta }$ = 0.04, 95% CRI = (−0.51, 0.59)). We are specifically interested in the interaction between intervening material and OT1H. Indeed, the analysis indicated that this interaction effect is present ($\hat{\beta }$ = 0.78, 95% CRI = (0.11, 1.44)). Model-based predictions reveal that when OT1H is absent, the probability of a contrastive continuation is 28.6% in the Intervening Absent condition and 36.1% in the Intervening Present condition. However, when OT1H is present, this probability rises to 91.3% in the Intervening Absent condition but only to 87.4% in the Intervening Present condition. This confirms that participants provide more contrastive continuations following OT1H in both conditions, but the effect is slightly attenuated when intervening material is present.

#### Bayes Factor Results.

**Figure 3.**
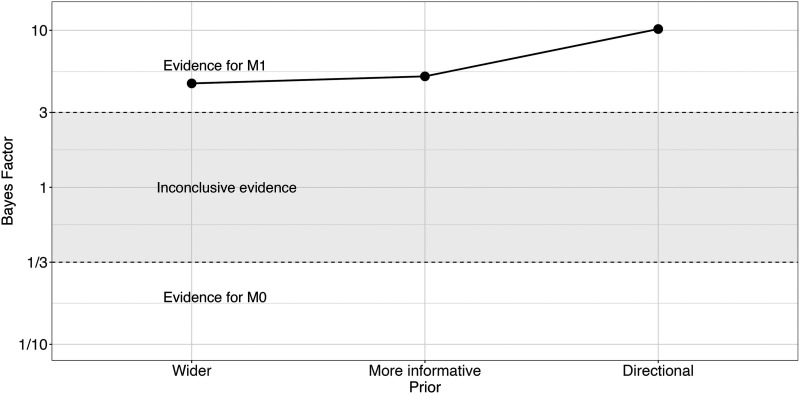
Bayes factor results for Model 1 over Model 0, Exp. 1.

To quantify the evidence in favour of the interaction effect, we conducted Bayes factor analyses. Figure 3 shows the results (BF_10_) for Model 1 (including the interaction term) and Model 0 (not including the interaction term). The Bayes factors showed moderate evidence in favor of the interaction effect in the expected direction (wider: BF_10_ = 5.1; informative: BF_10_ = 4.6; directional: BF_10_ = 10.2): the effect of OT1H is slightly larger in the Intervening Absent condition than in the Intervening Present condition.

#### Proportion of OTOH Usage.

The proportion of continuations that contained the explicit marker OTOH was not affected by presence of intervening material: OT1H was followed by OTOH specifically (rather than another contrastive connective such as *but*) in 58% of continuations in the Intervening Absent condition and 57% in the Intervening Present condition.

### Discussion Completion Study

Experiment 1 confirmed that OT1H elicits predictions of upcoming contrast: more contrastive continuations were provided for items containing the OT1H-cue compared to those that did not contain OT1H. The results also showed an effect of presence of intervening material on the facilitative effect of OT1H: the facilitative effect of OT1H was slightly weaker in the condition with intervening content compared to the condition without intervening content (85% contrastive continuations in the absence of intervening material vs. 80% contrastive continuations in the presence of intervening material).

In sum, the results from this offline continuation study indicate that comprehenders can use the OT1H-cue to generate expectations of OTOH, and that the presence of intervening material has a small effect on the maintenance of that expectation. Given that the materials were presented in an offline paradigm and the participants therefore had the opportunity to reread the materials and re-activate the prediction of OTOH. We further investigate the effect in online measures (see Experiments 2–4 below).

## EXPERIMENT 2: SELF-PACED READING

This experiment aims to test whether the effect of intervening material in offline processing replicates in online processing.

### Participants

A total of 166 native speakers of English (age range 20–79 years; mean age 40 years; 120 female) participated in this experiment. Data from four participants were excluded because they didn’t pass the comprehension question accuracy check. Participants were recruited via Prolific and received 2.50 GBP for their participation.

### Materials

As in Experiment 1, Experiment 2 was conducted in a 2 (OT1H-cue vs. no cue) × 2 (intervening absent vs. present, between-participants) design. The experimental stimuli consisted of the 16 experimental items used in the story continuation experiment, now including an OTOH-marked sentence whose content was intended to provide contrast2 (see (8)). The items were interspersed with 96 filler items for an unrelated study focusing on causal and list relations. None of these fillers contained contrastive discourse connectives or strong violations of expectations or anomalies. The stimuli were counterbalanced across four lists, with each story appearing in a different condition in each list. The participants were randomly assigned to one of the lists.

### Procedure

Participants were recruited via Prolific, after which they were directed to a website hosted by PC Ibex (Schwarz & Zehr, 2021), where they completed the moving window self-paced reading experiment. Items were initially displayed as a fixation cross. After pressing the space bar, participants were presented with a series of horizontal lines; the length of the lines corresponded to the length of the regions. By pressing the space bar on their keyboard, participants revealed the next region of the item. Items were presented non-cumulatively; when a new region was revealed, the previous region was again replaced by lines.

OT1H and OTOH were always presented as individual chunks, as well as the two words following OTOH. Moreover, OTOH never occurred as the first or final region of a line. Example (8) illustrates the spatial configuration of target stimuli on the screen, with slashes demarcating region.

(8)  Ronan is thinking about quitting his job / at the supermarket / after working there / for five years. / He thinks he could / get a more promising job / at a multinational, / because he studied accounting / in college. / On the other hand, / he has / a good / chance of becoming a manager / at the supermarket next year.

Once participants finished reading the item, they were presented with a verification statement after 25% of all items – that is, participants answered a total of 28 verification statements. Participants responded to the statement by pressing either ‘j’ for TRUE or ‘f’ for FALSE on their keyboard and received immediate feedback on accuracy. Mean accuracy on the verification statements was 89.5%.

At two occasions during the experiment, participants were instructed to take a short break. When they were ready to continue, participants clicked a “proceed” button at the bottom of the screen. The entire study lasted on average 20 minutes.

### Analysis Procedure

Prior to all analyses, extreme reading times longer than 5000 ms (2 cases) were removed. Outliers were removed by excluding reading times more than 2.5 times the standard deviation from a participant’s mean in a region (2.6% of the data points).

Reading times were compared on two regions: *On the other hand* (the critical region) and the two words following OTOH (the spillover region). The critical region is where a possible facilitative effect of OT1H is expected to be found: if readers anticipate OTOH based on OT1H, reading times of OTOH should be faster in the OT1H present condition than in the OTOH absent condition. We test whether this is dependent on the presence or absence of intervening material: if intervening material diminishes the facilitative effect of OT1H, we should see an interaction between OT1H and intervening material.

We fit Bayesian linear mixed-effects models with a lognormal family for each region separately. The analysis procedure was similar to that of Experiment 1: the deviation-coded variables for OT1H and for Intervening Material were included as fixed effect along with their interaction. The model also included random intercepts and slopes for participants and items. A centered covariate of trial order was included to account for any variance due to participants’ reading times speeding up over the course of experiment. We specified principled, weakly informative priors. Four chains were run using 10,000 iterations (including 2,000 warm-up).

We computed the Bayes factor for three types of informative priors on the parameter representing the interaction term: (1) a more informative prior *N*(0,0.05), (2) a wider prior *N*(0,0.1), and (3) a directional, empirical prior *N*(0.04, 0.02). To derive the empirical priors, we obtained model fits for data from an unpublished study using similar materials and an identical experimental paradigm in Dutch.

### Results

**Figure 4.**
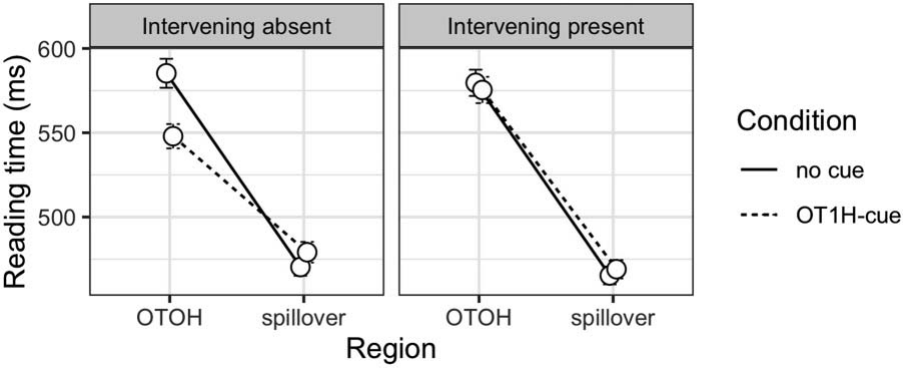
Mean reading times and error bars (SE) per region and condition, Exp. 2.

**Figure 5.**
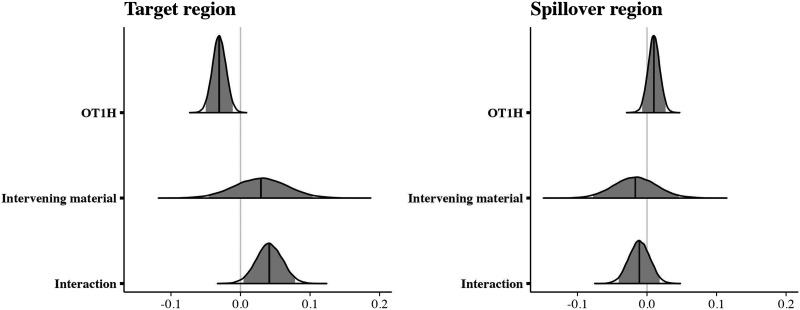
Posterior distributions for the comparisons between conditions, Exp. 2. The darker line shows the median posterior effect estimate; the light gray area indicates the 95% credible interval.

Figure 4 shows the reading times on the critical and spill-over region. Posterior distributions for the estimated parameters are depicted in Figure 5; the model results are shown in the Appendix (Table A2). The analysis indicated that reading of OTOH was overall faster when OT1H was present ($\hat{\beta }$ = −0.03, 95% CRI = (−0.05, −0.01)), suggesting a facilitative (main) effect of OT1H on OTOH. There was no main effect of intervening material ($\hat{\beta }$ = 0.03, 95% CRI = (−0.05, 0.10)). This was expected because in our design, when there was no OT1H cue present, there was no potential interference effect that the intervening material could pose. Therefore, we are specifically interested in the interaction between intervening material and OT1H. Indeed, the analysis indicated that this interaction effect is present at the target region ($\hat{\beta }$ = 0.04, 95% CRI = (0.00, 0.08)): when intervening material was absent, OT1H facilitated the processing of OTOH (follow-up model: $\hat{\beta }$ = −0.05, 95% CRI = (−0.09, −0.02)), but when intervening material was present, there was no facilitative effect of OT1H (follow-up model: $\hat{\beta }$ = −0.01, 95% CRI = (−0.03, 0.02)).

The results on the spillover region indicated no differences between any of the conditions; as is shown in Figure 5, the main effects as well as the interaction were centered around zero.

#### Bayes Factor Results.

**Figure 6.**
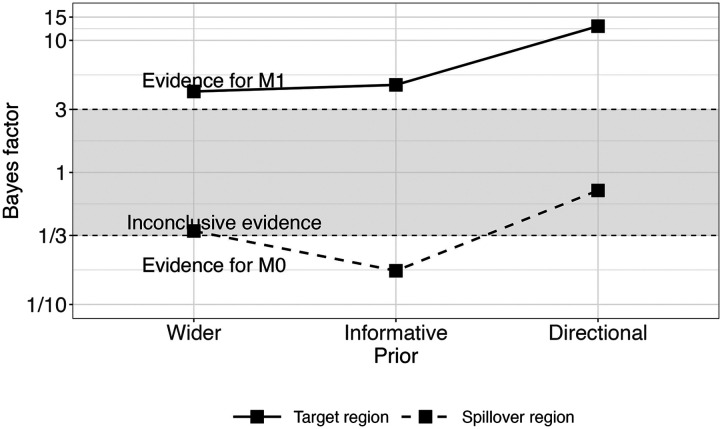
Bayes factor results for Model 1 over Model 0 per region, Exp. 2.

To quantify the evidence in favour of the interaction effect on the target region, we conducted Bayes factor analyses. Figure 6 shows the results (BF_10_) for Model 1 (including the interaction term) and Model 0 (not including the interaction term), separately for the target OTOH and spillover region. The Bayes factors showed moderate to strong evidence in favor of the interaction effect on the target OTOH region in the expected direction (wider: BF_10_ = 4.1; informative: BF_10_ = 4.6; directional: BF_10_ = 12.8): OTOH was read faster after OT1H, but only when intervening material was absent. For the spillover region, the Bayes factors showed anecdotal to moderate evidence for Model 0 (wider: BF_10_ = 0.36; informative: BF_10_ = 0.18; directional: BF_10_ = 0.73), which did not contain the interaction effect.

### Discussion Experiment 2

This study investigated whether the facilitative effect of OT1H on the processing of OTOH would persist or be diminished across intervening material. A Bayes factor analysis provided moderate to strong evidence that OT1H does facilitate the immediate processing of OTOH, but only in the absence of intervening material. In the presence of intervening material, the effect of OT1H is reduced, which indicates that the expectation elicited by OT1H has decayed due to the intervening material and is substantially weakened or lost at the point of encountering OTOH. These results therefore provide support for a memory-based account of language processing.

Before we can fully conclude that intervening material affects the facilitative effect of a discourse cue, we need to consider the possibility that the coarse-grained nature of self-paced reading data could influence the results. Perhaps more fine-grained reading time measures, as can be obtained with eye-tracking, could show an effect of OT1H on OTOH in the presence of intervening material. To further explore this, Experiment 3 was designed to complement the results from the self-paced reading study by investigating the effect of OT1H in an eye-tracking study.

If the finding in Experiment 2 of a lack of effect of OT1H in the presence of intervening material reflects a lack of sensitivity in our measure, then Experiment 3 may reveal that comprehenders can maintain predictions for OTOH across intervening material. If the Experiment 2 finding reflects a true deterioration of the prediction across intervening material, then a similar smaller effect of the OT1H-cue on OTOH in the presence of intervening material is expected in Experiment 3, showing the robustness of the patterns found in Experiment 2 even with a more sensitive measure.

## EXPERIMENT 3: EYE-TRACKING STUDY

An eye-tracking study was conducted to further investigate the effect of the presence of the marker OT1H on the processing of OTOH, in the presence of an intervening because-clause with a more precise method compared to SPR.

### Participants

A total of 90 native speakers of English (age range 18–36 years; mean age 22 years; 32 female, 1 non-binary)^2^ participated in this experiment. Participants were recruited from the University of Edinburgh and Saarland University communities and received monetary compensation for their participation. All participants had normal or corrected-to-normal vision. Data from ten participants could not be used due to problems with the eye-tracker; data from one participant were removed due to low accuracy on the verification statements. These data were removed before analysis. The final data file consists of data from 79 participants.

### Materials

The experiment was conducted in a 2 (OT1H-cue vs. no cue) × 2 (intervening absent vs. present) within-participants design. The experimental stimuli consisted of the same items used in Experiment 2. The items were interspersed with 84 filler items for an unrelated study focusing on the processing of causal relations, and for an unrelated study focusing on the processing of at-issueness. None of these fillers contained the discourse markers OT1H and OTOH, or strong anomalies.

The stimuli were counterbalanced across four lists, with each story appearing in a different condition in each list. The participants were randomly assigned to one of the lists. Items appeared in a fixed, pseudo-randomized order within lists^3^.

### Procedure

Participants were tested individually. They were seated at a distance of approximately 60 cm from the monitor and rested their head on a chin-rest. Eye movements were recorded with SR Research Eyelink 1000 at a sampling rate of 500 Hz. Half-way through the experiment, participants had a short break and completed two other tasks. Upon finishing these tasks, participants returned to the monitor, were recalibrated, and finished the eye tracking experiment. The experiment lasted approximately 50 minutes.

Each session started with an oral instruction, after which the eye-tracker was adjusted if necessary. A 9-point calibration procedure was then performed. Upon successful calibration, the experiment started with two practice trials. The participant was instructed to read the passage at a natural pace and press the space bar after reading the entire story. Before presentation, a fixation mark appeared at the position of the first word of the first sentence. Participants were instructed to fixate this mark, after which the story appeared. The stories were presented in their entirety on the screen. The critical region (“On the other hand,”) never appeared at the beginning or end of a line. A verification statement about the story followed 25% of the items to ensure that the participants read the passages carefully. Participants indicated whether the statement was correct or incorrect by pressing a button on a keyboard. Mean accuracy on the verification statements was 92%.

### Analysis Procedure

For analysis purposes the items were divided into three regions of interest, as illustrated in (9):

(9)  (…) / in college._*pre*−*critical*_ / On the other hand, *_criticalregion_* / he has *_spillover_* / (…)

Three reading time measures were computed: first pass duration, regression path duration and total reading time. First pass duration is the time spent in a region before moving on or looking back. This measure reflects the immediate processing difficulties a reader has when reading a region for the first time (Rayner, 1998). Regression path duration is the summed fixation duration from when the current region is first fixated until the eyes enter the next region on the right. This measure thus includes regressions to regions to the left of the current region. Regression path duration can be seen as reflecting the process of integrating the linguistic material with the previous context (Rayner, 1998). Total reading time is the total time spent in a region, including regressions to that region.

Prior to all analyses, skipped regions were treated as missing data. In all reading time measures, fixations shorter than 80 ms were removed. Outliers were removed by excluding reading times more than 2.5 standard deviations from the participant’s mean in a region (2.2% of the data points for the first pass duration, 4.1% for the regression path duration, and 2.3% for the total reading time duration).

We fit Bayesian linear mixed-effects models for each region and measure separately, using a similar approach as in Experiment 2. The variables for OT1H and for Intervening Material were deviation-coded and included as fixed effects along with their interaction. Participants and items were specified in the models as random effects, along with variance-covariance matrices. A centered covariate of trial order was included. We specified principled, weakly informative priors.

We computed the Bayes factor for two types of priors on the parameter representing the interaction effect: (1) a more informative prior *N*(0,0.05), and (2) a wider prior *N*(0,0.1). We also derived empirical, directional priors from the model fit reported in Experiment 2. Since Exp. 2 presented SPR data rather than eye-tracking data and only the target region and the spillover region, we restrict directional analyses to only these regions and to total fixation durations, which more closely approximate self-paced reading data (following Mertzen et al., 2024).

### Results

**Figure 7.**
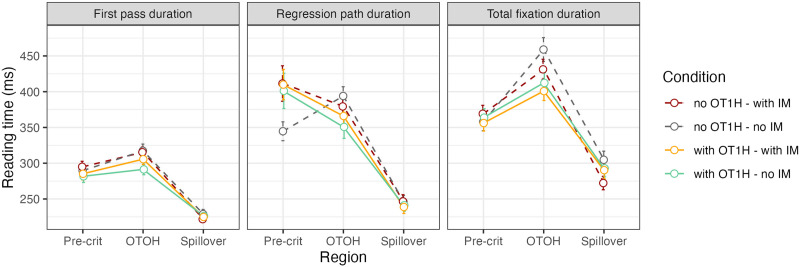
Mean reading times and error bars (SE) per measure, condition and region, Exp. 3. *IM* = intervening material.

**Figure 8.**
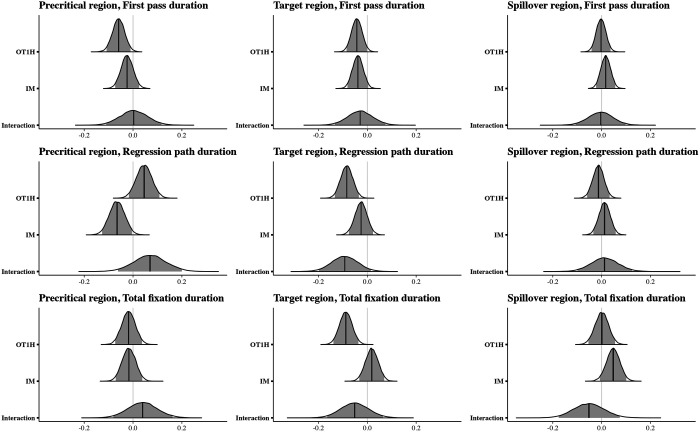
Posterior distributions for the comparisons between conditions, Exp. 3. The dark line shows the median posterior effect estimate; the light gray area indicates the 95% credible interval.

Figure 7 shows the reading times per region and measure. Posterior distributions for the estimated parameters are depicted in Figure 8; the model results are shown in the Appendix (Table A3).

The analysis indicated that the durations of all three reading measures on the target OTOH region were shorter when OT1H is present, suggesting a facilitative main effect of OT1H on OTOH (first pass: $\hat{\beta }$ = −0.04, 95% CRI = (−0.09, 0.00); regression path: $\hat{\beta }$ = −0.08, 95% CRI = (−0.13, −0.03); total fixation: $\hat{\beta }$ = −0.09, 95% CRI = (−0.14, −0.04)). We also found a main effect of intervening material in the first pass duration: reading times on the target region were shorter when no intervening material was present ($\hat{\beta }$ = −0.04, 95% CRI = (−0.08, 0.00)). The 95% CRI for these measures on the target region did not include 0. Crucially, we also observed an interaction effect on the target region in the regression path duration: the facilitative effect of OT1H on OTOH was smaller in the presence of intervening material ($\hat{\beta }$ = −0.09, 95% CRI = (−0.20, 0.01)). However, the 95% CRI for this effect did include 0. Bayes factor analyses will allow us to quantify the support for or against this effect.

The results on the precritical region indicated that first pass durations were shorter in the presence of OT1H ($\hat{\beta }$ = −0.06, 95% CRI = (−0.11, −0.01)). However, in the regression path duration, the direction of the effect appeared to be reversed ($\hat{\beta }$ = 0.05, 95% CRI = (−0.02, 0.11)), suggesting longer reading times on the precritical region. Note that, again, the 95% CRI for the regression path measure included 0. Crucially, the results showed no strong indication of an interaction effect on the precritical region.

The results on the spillover region indicated no differences between any of the conditions; as is shown in Figure 8, the main effects and interaction effect were centered around zero, with the exception of the intervening material effect in the total fixation duration ($\hat{\beta }$ = 0.05, 95% CRI = (−0.01, 0.10)). Zero was included in the 95% interval.

#### Bayes Factor Results.

**Figure 9.**
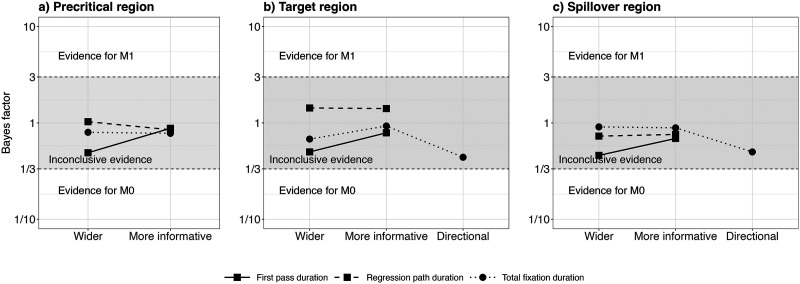
Bayes factor results for Model 1 over Model 0 per measure and region, Exp. 3.

To quantify the evidence in favour of or against the interaction effect, we conducted Bayes factor analyses. Figure 9 shows the results (BF_10_) for Model 1 (including the term for the interaction) and Model 0 (not including the term for the interaction), separately for the three measures and the precritical, critical and spillover region.

The Bayes factors showed anecdotal evidence in favor of an interaction effect on OTOH in the regression path duration (wider prior BF_10_ = 1.43, more informative prior: BF_10_ = 1.41).

In other words, these results indicate that intervening material might diminish a facilitative effect of OT1H on OTOH, specifically in the regression path duration. The other Bayes factors all either showed no evidence or anecdotal evidence against an interaction effect.

### Discussion Experiment 3

Experiment 3 was designed to test whether the facilitative effect of OT1H on processing of OTOH diminishes in the presence of intervening material, as the results from Experiment 2 indicated. The results from Experiment 3 indicated a main effect of OT1H facilitating the processing of OTOH. Further, the results showed anecdotal evidence that the facilitative effect might be diminished in the presence of intervening material, specifically in the regression path duration. However, the Bayes factors were not strong enough to be able to conclusively accept or reject this hypothesis.

Relating these results to the findings in Experiment 2, we can conclude that both support a main effect of OT1H on the processing of OTOH. This effect disappears in the presence of intervening material in Experiment 2’s self-paced reading paradigm, and appears to be reduced in Experiment 3’s eye-tracking paradigm. However, the results of this study do not allow us to draw any strong conclusions regarding how intervening materials affect discourse predictions in natural reading. We therefore zoom in on the effect of interest (i.e., the effect of OT1H presence/absence in the presence of intervening material) in a follow-up eye-tracking study. We test the same number of participants in a simpler design with 2 conditions (compared to the 2 × 2 design of Experiment 3), thus allowing us to preserve more power.

## EXPERIMENT 4: EYE-TRACKING STUDY, 2 CONDITIONS

The present eye-tracking experiment only uses two conditions (OT1H present vs. absent, in the presence of intervening material), to test whether the main effect of OT1H, which we have reliably found in experiments 2 and 3, can be found in a well-powered study with intervening material, or whether this main effect was always driven by the no-intervening-material condition.

If we find a Bayes factor substantially greater than 1 in this study, the evidence would favor the hypothesis that there is a facilitative effect of OT1H in the presence of intervening material. This would indicate that intervening material *does not* deteriorate the pre-activation of OTOH, thus supporting expectation-based accounts. A Bayes factor lower than 1 would indicate that the evidence favors the null hypothesis. This would indicate that intervening material *does* deteriorate the pre-activation of OTOH, thus supporting memory-based accounts. Finally, if we find a Bayes factor equal or very close to 1, we would find equal evidence for both hypotheses. This would indicate that we do not find clear evidence supporting a facilitative effect of OT1H in the presence of intervening material. In the context of the current study, such a result would thus support at least a slight deterioration of the pre-activation, which can be taken as support for memory-based models of processing.

### Participants

A total of 80 native speakers of English (age range 18–41 years; mean age 20 years; 63 female) participated in this experiment. Participants were recruited from the Lancaster University community and received course credit or monetary compensation for their participation. All participants had normal or corrected-to-normal vision. Data from 4 participants could not be used due to problems with the eye-tracker. These data were removed before analysis.

### Materials

The experimental stimuli consisted of the 16 experimental items with the intervening clause present, with the critical condition being the presence or absence of the OT1H-cue. The same 96 filler items used in Exp. 2 were included in this study. The stimuli were counterbalanced across two lists, with each story appearing in a different condition in each list. The participants were randomly assigned to one of the lists.

### Procedure

The procedure was identical to that of Experiment 3. Mean accuracy on the verification statements was 87.5%. Prior to all analyses, skipped regions were treated as missing data. In all reading time measures, fixations shorter than 80 ms were removed. Outliers were removed by excluding reading times more than 2.5 standard deviations from the participant’s mean in a region (2.0% of the data points for the first pass duration, 3.3% for the regression path duration, and 2.7% for the total reading time duration).

We fit Bayesian linear mixed-effects models for each region and measure separately. The variable for OT1H was deviation-coded (no OT1H: −0.5) and included as fixed effect. Participants and items were specified in the models as random effects, along with variance-covariance matrices. A centered covariate of trial order was included. We specified principled, weakly informative priors.

To calculate the Bayes factors, we used empirical priors derived from Experiment 3. We computed the Bayes factor for three types of priors on the parameter representing the condition: (1) a more informative prior *N*(0,0.05), (2) a wider prior *N*(0,0.1), and (3) a directional, empirical prior that differed per reading time measure (first pass duration: *N*(−0.03,0.05), regression path duration: *N*(−0.09,0.06), total fixation duration: *N*(−0.005,0.05)).

### Results

**Figure 10.**
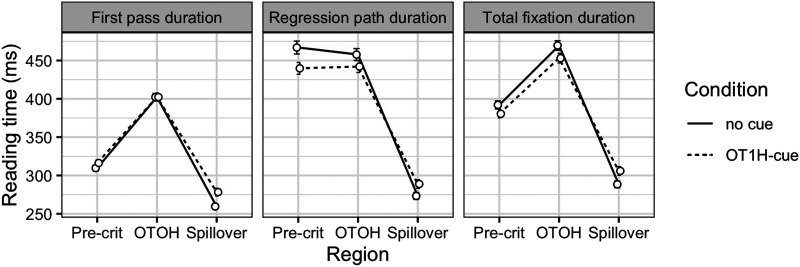
Mean reading times and error bars (SE) per measure, condition and region, Exp. 4.

**Figure 11.**
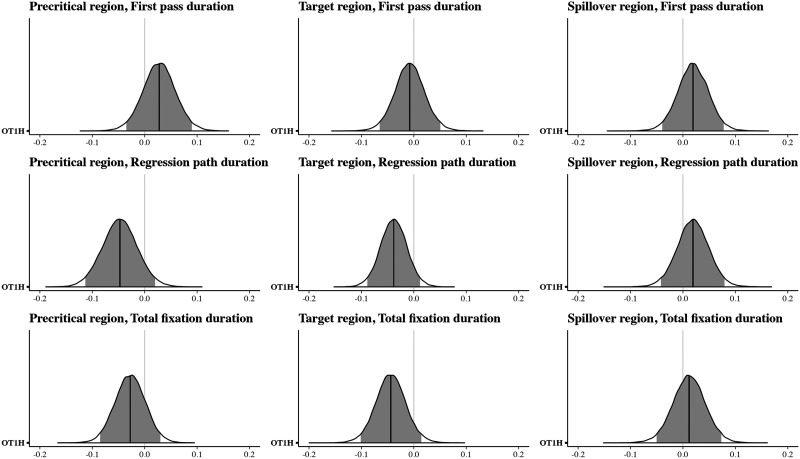
Posterior distributions for the comparisons between conditions, Exp. 4. The dark line shows the median posterior effect estimate; the light gray area indicates the 95% credible interval.

Figure 10 shows the reading times per region and measure. Posterior distributions for the estimated parameters are depicted in Figure 11; the model results are shown in the Appendix (Table A4). The analysis indicated that regression path durations and total fixation durations on OTOH were shorter when OT1H was present (regression path: $\hat{\beta }$ = −0.04, 95% CRI = (−0.09, 0.01); total fixation: $\hat{\beta }$ = −0.04, 95% CRI = (−0.10, 0.01)). However, the 95% CRI for both of these measures included 0 (see also Figure 11), meaning there is a chance that a difference in reading times in the presence and absence of OT1H is non-existent.

The results on the precritical region indicated that regression path durations of the two words preceding OTOH were shorter in the presence of OT1H ($\hat{\beta }$ = −0.05, 95% CRI = (−0.11, 0.02)). However, in the first pass duration, the direction of the effect appeared to be reversed, suggesting longer reading times on the precritical region ($\hat{\beta }$ = 0.03, 95% CRI = (−0.04, 0.09)). Note that, again, the 95% CRI for both of these measures included 0. The Bayes factor analyses will quantify the support for or against these observations.

The results on the spillover region indicated no differences between any of the conditions; as is shown in Figure 11 and Table A4, the main effects were centered around zero.

#### Bayes Factor Results.

**Figure 12.**
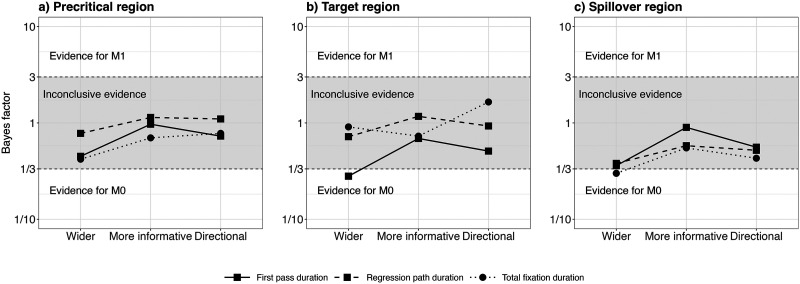
Bayes factor results for Model 1 over Model 0 per measure and region, Exp. 4.

To quantify the evidence in favour of or against the effect of OT1H, we conducted Bayes factor analyses. Figure 12 shows the results (BF_10_) for Model 1 (including the term for condition) and Model 0 (not including the term for condition), separately for the three regions and measures. At the precritical region, the Bayes factors showed either no evidence or anecdotal evidence against a facilitative effect of OT1H.

At the target region, the Bayes factor analysis for total fixation duration based on a directional prior (in favour of a facilitating effect of OT1H) was BF_10_ = 1.65. When using more neutral priors, the Bayes factor did not exceed 1 (wider prior: BF_10_ = 0.91; more informative prior: BF_10_ = 0.73), suggesting that the effect is sensitive to prior assumptions and that the evidence provided by our data is compatible with an account in which intervening material deteriorates the facilitative effect of OT1H.

Further, the Bayes factor analysis showed anecdotal to moderate evidence against a facilitative effect of OT1H in the first pass duration (wider: BF_10_ = 0.45, more informative: BF_10_ = 0.97, directional: BF_10_ = 0.73). Finally, in the spillover region, the Bayes factor analysis showed either no evidence or anecdotal to moderate evidence against a facilitative effect of OT1H in all measures (wider: BF_10_ = 0.42, more informative: BF_10_ = 0.70, directional: BF_10_ = 0.78).

### Discussion Experiment 4

Overall, the results of Experiment 4 do not provide supporting evidence for a facilitative effect of OT1H in the presence of intervening material on reading times. The Bayes factor analyses show only very weak support in a few cases with directional priors and often favor the null hypothesis: No facilitation effect across intervening material.

These results are in line with findings from the previous experiments: Experiment 2 showed a main effect of OT1H on OTOH as well as an interaction between the effect of OT1H and intervening material, which indicated that the facilitating effect of OT1H was not present in the conditions with intervening materials. Experiment 3 also found a main effect of OT1H, but there was only anecdotal evidence for the interaction with intervening material, motivating our more well-powered experiment 4. With 80 participants in a two-condition design, this study should have been able to detect a facilitation effect of OT1H, if such an effect exists. Taken together, these findings support the idea that the facilitative effect of OT1H is absent or much reduced when intervening material is present.

## GENERAL DISCUSSION AND CONCLUSION

The current study aimed to investigate whether comprehenders can maintain predictions of upcoming dependencies at the discourse level in the presence of intervening material. We manipulated the distance between the markers *On the one hand* and *On the other hand*, marking contrast1 and contrast2 respectively, by including an additional clause marked by *because*, presenting a reason for contrast1. The results of an offline continuation study showed that the marker OTOH was generally expected after an OT1H-marked contrast1, but that intervening material lead to a small reduction in contrastive completions. The results of a self-paced reading study indicated that intervening material had a stronger effect on online anticipations: OTOH was read faster when it was preceded by OT1H, but only when no intervening material was present between contrast1 and contrast2. To eliminate the possibility that this effect could be attributed to the coarse-grained nature of the self-paced reading paradigm, which could mask a possible effect during immediate processing or content integration, we conducted two follow-up eye-tracking experiments. Experiment 3 showed a clear facilitating main effect of OT1H on OTOH, and anecdotal evidence for an interaction, according to which that effect is reduced when intervening material is present. Experiment 4 found that the data do not support a facilitation of OT1H on OTOH when intervening material is present.

In sum, earlier studies (Scholman et al., 2024b; Schwab & Liu, 2020), as well as Experiments 2 and 3 (Exp. 4 cannot speak to this), did show a facilitated processing of the second marker when it was preceded by the first marker in the absence of intervening material, and we cannot find conclusive evidence for such an effect in conditions in which intervening material is present (Experiments 2 and 4; Experiment 3 shows a marginal effect). We therefore interpret the results as suggesting that the facilitative effects of a discourse cue do not persist across intervening distance between OT1H and OTOH (particularly in the first pass duration measure, which reflects early processing), or that those effects become so small that they are difficult to detect (in the regression path duration and total fixation duration, which reflect integration).

The evidence presented in the current study is therefore in line with evidence from the decay and interference literature, as well as the locality effect literature. It remains an open question what underlying factor can explain the diminished facilitative effect of OT1H in the presence of intervening material. One possibility is that the intervening material causes decay of the anticipation, simply because the distance between the pair of markers is increased (i.e., the anticipation is affected by temporal interference). Another option is that the content of the intervening material gives rise to new, competing expectations of upcoming discourse, which interfere with the original expectation of contrast and distract the comprehender (i.e., the anticipation is affected by linguistic interference). It is also possible that neither decay nor interference can explain the current results. It might be that linguistic prediction is disengaged when it is less beneficial or more costly to maintain (see also Martin et al., 2020). For example, Wlotko and Federmeier (2015) found a lack of prediction-related effects on the N400 when the rate of incoming words during sentence reading was very high, suggesting that individuals did not engage the same mechanisms as when the rate was slower. In the current study, the prediction of OTOH might have been less beneficial to maintain once the reader encountered an intervening clause, and the reader may thus have reduced the activation of no longer relevant material (the OT1H cue).

Our results cannot distinguish between the decay versus interference explanations since, in our design, the intervening material always consisted of a subordinate clause with ten words marked by *because*. Follow-up studies could focus on the distinction between decay and interference with respect to discourse predictions. For example, one could vary the length of the intervening clause (five words versus ten words), the type of intervening clause (full sentence versus subordinate clause), the type of discourse content (a causal relation versus a more complex concessive relation), or the type of semantic content (non-sensical strings versus a normal clause). One could also manipulate the content of the intervening material to interfere with the retrieval of the original OT1H more or less strongly, for example, by comparing an intervening sentence that strengthens the prediction of a contrast relation (e.g., a sentence signaling contrast, such as *This is only one option*) to a sentence that generates other, competing discourse predictions (e.g., a sentence with an implicit causality verb, which gives rise to causal expectations).

Our findings shed new light on the earlier findings that comprehenders can build and maintain discourse predictions over multi-sentence passages. Scholman et al. (2017) tested integration of contrast2 after a plausibly expectation-satisfying contrast had been provided in intervening material, and found that readers were affected by this material. In other words, the results indicated that readers had integrated the intervening material with the contrast dependency, and surprisal affected processing when they encountered OTOH and realized that the contrast dependency expectation was, in fact, not yet satisfied. Such surprisal did not occur when the intervening material did not provide a plausible contrast. Crucially, that result suggests that readers were able to generate predictions of discourse dependencies during online processing, and maintain these predictions across intervening material. However, Scholman et al. (2017) did not test the prediction of discourse markers by manipulating presence/absence of OT1H, and so that study cannot speak to expectation-driven effects specifically (but rather to discourse-building effects). The results from the current study do speak to such effects, and suggest that intervening material affects discourse predictions.

How, then, can these findings be integrated into a plausible account of discourse processing and expectations? Perhaps the predictions that comprehenders made in the two studies function on different levels: one involving the generation of discourse structural dependencies, and one involving the generation of lexical predictions. Scholman et al. (2017) tested the prediction of a discourse dependency, with intervening material either satisfying the prediction or not. In contrast, the current study tested the facilitative effect of OT1H on the processing of a specific lexical marker (OTOH) in the presence of intervening material. It is possible that the generation of an upcoming contrast dependency was generated and maintained in the current study, but the prediction of the specific lexical marker of this dependency deteriorated. Scholman et al. (2017) thus found evidence for robust expectations of discourse dependencies, while the current study showed that predictions of upcoming markers are present, but are less persistent than dependency expectations.

Such an interpretation of the findings of both studies would converge with evidence from the field of structural (syntactic) priming. Structural priming effects have been found in paradigms where participants listen to or read a prime sentence, such as a picture description, and subsequently produce a target sentence, oftentimes describing a different picture with a given verb. For example, participants might be presented with material that biases them toward producing a double object (DO: *Amanda gave Mick a box.*) or a prepositional object (PO: *Amanda gave a box to Mick.*) construction. Subsequently, participants are asked to produce target sentences that allow completion as either a DO or PO construction (*Emma sent…*). Participants are shown to favor the reuse of the syntactic structure of the prime sentence in their production of a target sentence. In addition to this established structural priming effect, previous work has also suggested the existence of a *lexical boost* effect (Pickering & Branigan, 1998), which occurs when the verb is repeated between the prime sentence and the target sentence. In such cases, the structural priming effects tend to be larger. There is evidence that the structural priming effect can (but does not always) persist over several intervening sentences (Branigan et al., 1999, 2000; Bock & Griffin, 2000; Hartsuiker et al., 2008). However, the lexical boost effect decays much more rapidly (Hartsuiker et al., 2008). The difference between the durations of the effects suggests that they operate on different levels: syntactic structure primes are more persistent than lexical primes. Drawing an analogy between these results and the current study, it is possible that the effect of OT1H on the processing of OTOH is of a lexical and fleeting nature, while the effect of type of intervening material on discourse predictions (as tested in Scholman et al., 2017) is of a structural and persistent nature.^4^

Alternatively, we have to consider the possibility that the facilitative effect of OT1H on OTOH is not an effect of prediction at all, but rather of retrieval of the OT1H cue. In other words, it is not the expectation of OTOH that deteriorated, but rather the trace of OT1H that was difficult to retrieve from memory (owing to decay or interference). In this case, the results should be interpreted as reflecting a “backward-looking” cost (i.e., the cost of retrieving and integrating previously processed OT1H with the incoming OTOH) rather than a “forward-looking cost” (i.e., the cost of updating predictions that are incompatible with the current material being processed) (Demberg & Keller, 2008). The results of the current study do not provide clear evidence on which phenomenon is exactly at play, although recent accounts argue that prediction and retrieval are, in fact, functionally the same (see also Ferreira & Chantavarin, 2018; Huettig et al., 2022; Schoknecht et al., 2022).

For future research, it would be interesting to explore to what extent the effect of OT1H as a signal and the effect of the intervening material are affected by the characteristics of the individual reader. Comprehension of connectives and sensitivity to relational markers are associated with various potentially influencing factors at the individual level, most notably linguistic experience. For example, prior work has shown that linguistic experience is relevant for connective comprehension (Scholman et al., 2024a; Wetzel et al., 2020; Zufferey & Gygax, 2020) as well as sensitivity to relational signals (Scholman et al., 2020). Similarly, the effect of intervening material in dependency locality studies has been shown to vary between comprehenders, with working memory capacity being a relevant factor (Nicenboim et al., 2015, 2016). Therefore, it is likely that the facilitative effect of OT1H and the disruptive effect of the intervening material on the processing of OTOH differ between comprehenders, depending on various reader characteristics such as their sensitivity to the statistical correlation between OT1H and OTOH and their cognitive capacities.

In sum, the current study provided more insight into the robustness of predictions by studying how the facilitative effect of the discourse marker OT1H on the processing of OTOH is affected by the presence of intervening material. Our results add to evidence that comprehenders do generate discourse expectations at the discourse level, contrary to an account in which discourse expectation maintenance could be considered ineffective due to the extensive flexibility and optionality at play at the discourse level. However, and crucially, the current work indicates that discourse expectations deteriorate rapidly across an intervening clause during real-time processing. This supports memory-based models of processing, suggesting that discourse processing becomes more difficult with additional material.

## Funding Information

This work was supported by the Deutsche Forschungsgemeinschaft, Funder ID: 10.13039/501100001659, Grant Number: SFB1102: Information Density and Linguistic Encoding.

## Acknowledgments

We are grateful to Marian Marchal, Benedek Bartha, Sinead Cleary, Jiong Xiao, Markéta Ceháková, Brianna Lehman, and Domenik Mayer for their help in collecting and processing the data for Exp. 3, and Kate Cain, Liam Blything, Gill Francey, and Nicola Currie for their help in collecting the data for Exp. 4.

## Data Availability

The materials, code and anonymized data for all studies are available online: https://osf.io/dh48q.

## Notes

^1^ In the majority of OT1H-marked instances (60%), OT1H and OTOH occurred in the same sentence. In 15% of the instances, OT1H and OTOH occurred in adjacent sentences.^2^ Due to a technical error, demographic information of 31 participants is unavailable.^3^ This differed from the other studies, in which items were presented in a random order. The change in this study was done to accommodate the unrelated studies that functioned as filler items for the current study. Trial order is included in all models in all experiments as covariate, thus accounting for any variance.^4^ The structural priming and lexical boost effects do, of course, relate to different linguistic mechanisms (syntax and production) than the phenomenon under investigation in the current study (discourse and comprehension). Moreover, the structural priming and lexical boost effects are different in nature to prediction effects, since the prime is not taken to lead to predictions that the structure will come again; that is, there is not necessarily any top-down predictive activation, but only a bottom-up integration effect in priming.

## APPENDIX

**Table A1.** Coefficients from Bayesian mixed-effects model for the effects of the conditions, Exp. 1.

	Posterior $\hat{\beta }$	CRI low.	CRI up.
Intercept	0.71	0.27	1.18
OT1H	2.95	2.18	3.57
Intervening Material	0.04	−0.51	0.59
Interaction	0.78	0.11	1.44

**Table A2.** Coefficients from Bayesian mixed-effects model for the effects of conditions for each region, Exp. 2.

	Target			Spillover		
	Posterior $\hat{\beta }$	CRI low.	CRI up.	Posterior $\hat{\beta }$	CRI low.	CRI up.
(Intercept)	6.30	6.26	6.32	6.12	6.08	6.15
Trial order	0.00	0.00	0.00	0.00	0.00	0.00
OT1H	−0.03	−0.05	−0.01	0.01	−0.01	0.03
Intervening material	0.03	−0.05	0.10	−0.02	−0.08	0.05
Interaction	0.04	0.00	0.08	−0.01	−0.04	0.02

**Table A3.** Coefficients from Bayesian mixed-effects model for the effect of condition for each region and each measure, Exp. 3.

	Precritical			Target			Spillover		
	Post. $\hat{\beta }$	CRI low.	CRI up.	Post. $\hat{\beta }$	CRI low.	CRI up.	Post. $\hat{\beta }$	CRI low.	CRI up.
First pass duration									
(Intercept)	5.64	5.49	5.79	5.71	5.63	5.81	5.34	5.22	5.46
Trial order	−0.10	−0.24	0.04	−0.08	−0.15	−0.01	−0.03	−0.14	0.09
OT1H	−0.06	−0.11	−0.01	−0.04	−0.09	0.00	0.00	−0.04	0.04
IM	−0.02	−0.07	0.02	−0.04	−0.08	0.00	0.02	−0.02	0.06
Interaction	0.00	−0.11	0.12	−0.03	−0.13	0.07	0.00	−0.11	0.10

Regression path duration									
(Intercept)	5.87	5.70	6.03	5.89	5.78	6.03	5.37	5.25	5.49
Trial order	−0.09	−0.25	0.07	−0.10	−0.20	−0.01	−0.01	−0.12	0.10
OT1H	0.05	−0.02	0.11	−0.08	−0.13	−0.03	−0.01	−0.06	0.03
IM	−0.07	−0.13	0.00	−0.03	−0.08	0.02	0.01	−0.03	0.06
Interaction	0.07	−0.13	0.00	−0.09	−0.20	0.01	0.01	−0.10	0.12

Total fixation duration									
(Intercept)	5.91	5.74	6.08	6.05	5.94	6.16	5.56	5.40	5.72
Trial order	−0.18	−0.35	−0.02	−0.15	−0.24	−0.05	−0.06	−0.22	0.09
OT1H	−0.02	−0.07	0.04	−0.09	−0.14	−0.04	0.00	−0.05	0.05
IM	−0.02	−0.07	0.04	0.02	−0.03	0.07	0.05	−0.01	0.10
Interaction	0.04	−0.08	0.16	−0.05	−0.16	0.06	−0.05	−0.18	0.07

**Table A4.** Coefficients from Bayesian mixed-effects model for the effect of condition for each region and each measure, Exp. 4.

	Precritical			Target			Spillover		
	Post. $\hat{\beta }$	CRI low.	CRI up.	Post. $\hat{\beta }$	CRI low.	CRI up.	Post. $\hat{\beta }$	CRI low.	CRI up.
First pass duration									
(Intercept)	5.63	5.54	5.72	5.91	5.84	5.97	5.46	5.37	5.54
Trial order	0.00	0.00	0.00	0.00	0.00	0.00	0.00	0.00	0.00
OT1H	0.03	−0.04	0.09	−0.01	−0.07	0.05	0.02	−0.04	0.08

Regression path duration									
(Intercept)	5.93	5.83	6.04	5.89	5.91	6.06	5.48	5.39	5.57
Trial order	0.00	0.00	0.00	0.00	0.00	0.00	0.00	0.00	0.00
OT1H	−0.05	−0.11	0.02	−0.04	−0.09	0.01	0.02	−0.04	0.08

Total fixation duration									
(Intercept)	5.82	5.72	5.93	6.03	5.85	6.10	5.54	5.44	5.63
Trial order	0.00	0.00	0.00	0.00	0.00	0.00	0.00	0.00	0.00
OT1H	−0.03	−0.09	0.03	−0.04	−0.10	0.01	0.01	−0.05	0.07

## References

[bib1] Asr, F. T., & Demberg, V. (2020). Interpretation of discourse connectives is probabilistic: Evidence from the study of but and although. Discourse Processes, 57, 376–399. 10.1080/0163853X.2019.1700760

[bib2] Bartek, B., Lewis, R. L., Vasishth, S., & Smith, M. R. (2011). In search of on-line locality effects in sentence comprehension. Journal of Experimental Psychology: Learning, Memory, and Cognition, 37, 1178–1198. 10.1037/a0024194, 21707210

[bib3] Barthel, M., Tomasello, R., & Liu, M. (2022). Online comprehension of conditionals in context: A self-paced reading study on wenn (‘if’) versus nur wenn (‘only if’) in German. Linguistics Vanguard, 8, 371–381. 10.1515/lingvan-2021-0083

[bib4] Berman, M. G., Jonides, J., & Lewis, R. L. (2009). In search of decay in verbal short-term memory. Journal of Experimental Psychology: Learning, Memory, and Cognition, 35, 317–333. 10.1037/a0014873, 19271849 PMC3980403

[bib5] Bock, K., & Griffin, Z. M. (2000). The persistence of structural priming: Transient activation or implicit learning? Journal of Experimental Psychology: General, 129, 177–192. 10.1037/0096-3445.129.2.177, 10868333

[bib6] Branigan, H. P., Pickering, M. J., & Cleland, A. A. (1999). Syntactic priming in written production: Evidence for rapid decay. Psychonomic Bulletin & Review, 6, 635–640. 10.3758/BF03212972, 10682206

[bib7] Branigan, H. P., Pickering, M. J., Stewart, A. J., & McLean, J. F. (2000). Syntactic priming in spoken production: Linguistic and temporal interference. Memory & Cognition, 28, 1297–1302. 10.3758/BF03211830, 11219957

[bib8] Brown, J. (1958). Some tests of the decay theory of immediate memory. Quarterly Journal of Experimental Psychology, 10, 12–21. 10.1080/17470215808416249 PMC4241183 24853316

[bib9] Bürkner, P.-C. (2017). brms: An R package for Bayesian multilevel models using Stan. Journal of Statistical Software, 80, 1–28. 10.18637/jss.v080.i01

[bib10] Campanelli, L., Van Dyke, J. A., & Marton, K. (2018). The modulatory effect of expectations on memory retrieval during sentence comprehension. In Proceedings of the 40th Annual Conference of the Cognitive Science Society (CogSci) (pp. 1436–1441).

[bib11] Crible, L. (2021). Negation cancels discourse-level processing differences: Evidence from reading times in concession and result relations. Journal of Psycholinguistic Research, 50, 1283–1308. 10.1007/s10936-021-09802-2, 34363178

[bib12] Demberg, V., & Keller, F. (2008). Data from eye-tracking corpora as evidence for theories of syntactic processing complexity. Cognition, 109, 193–210. 10.1016/j.cognition.2008.07.008, 18930455

[bib13] Dery, J. E., & Koenig, J.-P. (2015). A narrative-expectation-based approach to temporal update in discourse comprehension. Discourse Processes, 52, 559–584. 10.1080/0163853X.2014.966293

[bib14] Ferreira, F., & Chantavarin, S. (2018). Integration and prediction in language processing: A synthesis of old and new. Current Directions in Psychological Science, 27, 443–448. 10.1177/0963721418794491, 31130781 PMC6530918

[bib15] Ferreira, F., & Henderson, J. M. (1991). Recovery from misanalyses of garden-path sentences. Journal of Memory and Language, 30, 725–745. 10.1016/0749-596X(91)90034-H

[bib16] Fine, A. B., Jaeger, T. F., Farmer, T. A., & Qian, T. (2013). Rapid expectation adaptation during syntactic comprehension. PLoS One, 8, e77661. 10.1371/journal.pone.0077661, 24204909 PMC3813674

[bib17] Gibson, E. (1998). Linguistic complexity: Locality of syntactic dependencies. Cognition, 68, 1–76. 10.1016/S0010-0277(98)00034-1, 9775516

[bib18] Grodner, D., & Gibson, E. (2005). Consequences of the serial nature of linguistic input for sentenial complexity. Cognitive Science, 29, 261–290. 10.1207/s15516709cog0000_7, 21702774

[bib19] Hale, J. (2001). A probabilistic Earley parser as a psycholinguistic model. In Proceedings of the Second Meeting of the North American Chapter of the Association for Computational Linguistics on Language Technologies (pp. 1–8). Pittsburgh, PA.

[bib20] Hartsuiker, R. J., Bernolet, S., Schoonbaert, S., Speybroeck, S., & Vanderelst, D. (2008). Syntactic priming persists while the lexical boost decays: Evidence from written and spoken dialogue. Journal of Memory and Language, 58, 214–238. 10.1016/j.jml.2007.07.003

[bib21] Hoek, J., Rohde, H., Evers-Vermeul, J., & Sanders, T. J. M. (2021). Expectations from relative clauses: Real-time coherence updates in discourse processing. Cognition, 210, 104581. 10.1016/j.cognition.2020.104581, 33497917

[bib22] Huettig, F., Audring, J., & Jackendoff, R. (2022). A parallel architecture perspective on pre-activation and prediction in language processing. Cognition, 224, 105050. 10.1016/j.cognition.2022.105050, 35398592

[bib23] Husain, S., Vasishth, S., & Srinivasan, N. (2014). Strong expectations cancel locality effects: Evidence from hindi. PLoS One, 9, e100986. 10.1371/journal.pone.0100986, 25010700 PMC4091936

[bib24] Köhne-Fuetterer, J., Drenhaus, H., Delogu, F., & Demberg, V. (2021). The online processing of causal and concessive discourse connectives. Linguistics, 59, 417–448. 10.1515/ling-2021-0011

[bib25] Konieczny, L. (2000). Locality and parsing complexity. Journal of Psycholinguistic Research, 29, 627–645. 10.1023/A:1026528912821, 11196066

[bib26] Lee, M. D., & Wagenmakers, E.-J. (2014). Bayesian cognitive modeling: A practical course. Cambridge University Press. 10.1017/CBO9781139087759

[bib27] Levy, R. (2008). Expectation-based syntactic comprehension. Cognition, 106, 1126–1177. 10.1016/j.cognition.2007.05.006, 17662975

[bib28] Levy, R. P., & Keller, F. (2013). Expectation and locality effects in German verb-final structures. Journal of Memory and Language, 68, 199–222. 10.1016/j.jml.2012.02.005, 24558294 PMC3928089

[bib29] Lewandowski, D., Kurowicka, D., & Joe, H. (2009). Generating random correlation matrices based on vines and extended onion method. Journal of Multivariate Analysis, 100, 1989–2001. 10.1016/j.jmva.2009.04.008

[bib30] Lewandowsky, S., & Oberauer, K. (2008). The word-length effect provides no evidence for decay in short-term memory. Psychonomic Bulletin & Review, 15, 875–888. 10.3758/PBR.15.5.875, 18926980

[bib31] Lewandowsky, S., Oberauer, K., & Brown, G. D. A. (2009). No temporal decay in verbal short-term memory. Trends in Cognitive Sciences, 13, 120–126. 10.1016/j.tics.2008.12.003, 19223224

[bib32] Lewis, R. L., Vasishth, S., & Van Dyke, J. A. (2006). Computational principles of working memory in sentence comprehension. Trends in Cognitive Sciences, 10, 447–454. 10.1016/j.tics.2006.08.007, 16949330 PMC2239011

[bib33] Martin, J. D., Shipstead, Z., Harrison, T. L., Redick, T. S., Bunting, M., & Engle, R. W. (2020). The role of maintenance and disengagement in predicting reading comprehension and vocabulary learning. Journal of Experimental Psychology: Learning, Memory, and Cognition, 46, 140–154. 10.1037/xlm0000705, 31169403

[bib34] McKone, E. (1995). Short-term implicit memory for words and nonwords. Journal of Experimental Psychology: Learning, Memory, and Cognition, 21, 1108–1126. 10.1037/0278-7393.21.5.1108

[bib35] McKone, E. (1998). The decay of short-term implicit memory: Unpacking lag. Memory & Cognition, 26, 1173–1186. 10.3758/BF03201193, 9847544

[bib36] Mertzen, D., Laurinavichyute, A., Dillon, B. W., Engbert, R., & Vasishth, S. (2024). Crosslinguistic evidence against interference from extra-sentential distractors. Journal of Memory and Language, 137, 104514. 10.1016/j.jml.2024.104514

[bib37] Nakatani, K., & Gibson, E. (2010). An on-line study of japanese nesting complexity. Cognitive Science, 34, 94–112. 10.1111/j.1551-6709.2009.01067.x, 21564207

[bib38] Nicenboim, B., Logačev, P., Gattei, C., & Vasishth, S. (2016). When high-capacity readers slow down and low-capacity readers speed up: Working memory and locality effects. Frontiers in Psychology, 7, 280. 10.3389/fpsyg.2016.00280, 27014113 PMC4782223

[bib39] Nicenboim, B., Vasishth, S., Gattei, C., Sigman, M., & Kliegl, R. (2015). Working memory differences in long-distance dependency resolution. Frontiers in Psychology, 6, 312. 10.3389/fpsyg.2015.00312, 25852623 PMC4369666

[bib40] Oberauer, K., & Lewandowsky, S. (2014). Further evidence against decay in working memory. Journal of Memory and Language, 73, 15–30. 10.1016/j.jml.2014.02.003

[bib41] Peterson, L., & Peterson, M. J. (1959). Short-term retention of individual verbal items. Journal of Experimental Psychology, 58, 193–198. 10.1037/h0049234, 14432252

[bib42] Pickering, M. J., & Branigan, H. P. (1998). The representation of verbs: Evidence from syntactic priming in language production. Journal of Memory and Language, 39, 633–651. 10.1006/jmla.1998.2592

[bib43] Pusse, F., Sayeed, A., & Demberg, V. (2016). LingoTurk: Managing crowdsourced tasks for psycholinguistics. In Proceedings of the North American Chapter of the Association for Computational Linguistics: Human Language Technologies (NAACL-HLT) (pp. 57–61). San Diego, CA.

[bib44] Rayner, K. (1998). Eye movements in reading and information processing: 20 years of research. Psychological Bulletin, 124, 372–422. 10.1037/0033-2909.124.3.372, 9849112

[bib45] Reitman, J. S. (1974). Without surreptitious rehearsal, information in short-term memory decay. Journal of Verbal Learning and Verbal Behavior, 13, 365–377. 10.1016/S0022-5371(74)80015-0

[bib46] Ricker, T. J., Vergauwe, E., & Cowan, N. (2016). Decay theory of immediate memory: From Brown (1958) to today (2014). Quarterly Journal of Experimental Psychology, 69, 1969–1995. 10.1080/17470218.2014.914546, 24853316 PMC4241183

[bib47] Rohde, H., & Horton, W. S. (2014). Anticipatory looks reveal expectations about discourse relations. Cognition, 133, 667–691. 10.1016/j.cognition.2014.08.012, 25247235

[bib48] Safavi, M. S., Husain, S., & Vasishth, S. (2016). Dependency resolution difficulty increases with distance in persian separable complex predicates: Evidence for expectation and memory-based accounts. Frontiers in Psychology, 7, 403. 10.3389/fpsyg.2016.00403, 27064660 PMC4812816

[bib49] Schoknecht, P., Roehm, D., Schlesewsky, M., & Bornkessel-Schlesewsky, I. (2022). The interaction of predictive processing and similarity-based retrieval interference: An erp study. Language, Cognition and Neuroscience, 37, 883–901. 10.1080/23273798.2022.2026421

[bib50] Scholman, M. C. J., Demberg, V., & Sanders, T. J. M. (2020). Individual differences in expecting coherence relations: Exploring the variability in sensitivity to contextual signals in discourse. Discourse Processes, 57, 844–861. 10.1080/0163853X.2020.1813492

[bib51] Scholman, M. C. J., Rohde, H., & Demberg, V. (2017). “On the one hand” as a cue to anticipate upcoming discourse structure. Journal of Memory and Language, 97, 47–60. 10.1016/j.jml.2017.07.010

[bib52] Scholman, M., Marchal, M., & Demberg, V. (2024a). Connective comprehension in adults: The influence of lexical transparency, frequency, and individual differences. Discourse Processes, 61, 381–403. 10.1080/0163853X.2024.2325262, 39193317 PMC11346385

[bib53] Scholman, M., Rohde, H., & Demberg, V. (2024b). Facilitation of lexical form or discourse relation: Evidence from contrastive pairs of discourse markers. Glossa Psycholinguistics, 3. 10.5070/G60111353

[bib54] Schwab, J., & Liu, M. (2020). Lexical and contextual cue effects in discourse expectations: Experimenting with German ‘zwar…aber’ and English ‘true/sure…but’. Dialogue & Discourse, 11, 74–109. 10.5087/dad.2020.203

[bib55] Schwab, J., Xiang, M., & Liu, M. (2022). Antilocality effect without head-final dependencies. Journal of Experimental Psychology: Learning, Memory, and Cognition, 48, 446–463. 10.1037/xlm0001079, 35084924

[bib56] Schwarz, F., & Zehr, J. (2021). Tutorial: Introduction to pcibex–an open-science platform for online experiments: Design, data-collection and code-sharing. In Proceedings of the Annual Meeting of the Cognitive Science Society (Vol. 43, pp. 15–16).

[bib57] Staub, A., & Clifton, C., Jr. (2006). Syntactic prediction in language comprehension: Evidence from either…or. Journal of Experimental Psychology: Learning, Memory, and Cognition, 32, 425–436. 10.1037/0278-7393.32.2.425, 16569157 PMC1479855

[bib58] Stone, K., von der Malsburg, T., & Vasishth, S. (2020). The effect of decay and lexical uncertainty on processing long-distance dependencies in reading. PeerJ, 8, e10438. 10.7717/peerj.10438, 33362963 PMC7750004

[bib59] Underwood, B. J., & Keppel, G. (1962). One-trial learning? Journal of Memory and Language, 1, 1–13. 10.1016/S0022-5371(62)80013-9

[bib60] Van Dyke, J. A., & Lewis, R. L. (2003). Distinguishing effects of structure and decay on attachment and repair: A cue-based parsing account of recovery from misanalyzed ambiguities. Journal of Memory and Language, 49, 285–316. 10.1016/S0749-596X(03)00081-0

[bib61] Vasishth, S., & Lewis, R. L. (2006). Argument-head distance and processing complexity: Explaining both locality and antilocality effects. Language, 767–794. 10.1353/lan.2006.0236

[bib62] Vasishth, S., Mertzen, D., Jäger, L. A., & Gelman, A. (2018). The statistical significance filter leads to overoptimistic expectations of replicability. Journal of Memory and Language, 103, 151–175. 10.1016/j.jml.2018.07.004

[bib63] Wetzel, M., Zufferey, S., & Gygax, P. (2020). Second language acquisition and the mastery of discourse connectives: Assessing the factors that hinder L2-learners from mastering French connectives. Languages, 5, 35. 10.3390/languages5030035

[bib64] Wlotko, E. W., & Federmeier, K. D. (2015). Time for prediction? the effect of presentation rate on predictive sentence comprehension during word-by-word reading. Cortex, 68, 20–32. 10.1016/j.cortex.2015.03.014, 25987437 PMC4832567

[bib65] Xiang, M., & Kuperberg, G. (2015). Reversing expectations during discourse comprehension. Language, Cognition and Neuroscience, 30, 648–672. 10.1080/23273798.2014.995679, 25914891 PMC4405243

[bib66] Yi, E., & Koenig, J.-P. (2021). Grammar modulates discourse expectations: Evidence from causal relations in English and Korean. Language and Cognition, 13, 99–127. 10.1017/langcog.2020.29

[bib67] Zufferey, S., & Gygax, P. (2020). “Roger broke his tooth. However, he went to the dentist”: Why some readers struggle to evaluate wrong (and right) uses of connectives. Discourse Processes, 57, 184–200. 10.1080/0163853X.2019.1607446

